# Multidisciplinary management of orthopaedic trauma – are we adhering to the guidelines?

**DOI:** 10.1308/rcsann.2024.0048

**Published:** 2024-07-31

**Authors:** K Hutchinson, CB Bretherton, A Gmati, B Handley, Get It Right First Time (GIRFT) Collaborators

**Affiliations:** ^1^ Croydon University Hospital, UK; ^2^ Queen Mary University, UK; ^3^ West Midlands, UK; ^4^ John Radcliffe Hospital, UK

**Keywords:** Orthopaedic trauma, Frailty, MDT

## Abstract

**Introduction:**

A multidisciplinary team (MDT) approach to polytrauma patients minimises morbidity and mortality. This project assesses the extent to which British Orthopaedic Association Standards for Trauma guidelines for the management of the frail Orthopaedic patient are currently being met.

**Methods:**

A retrospective analysis was performed of all Trauma and Orthopaedic patients in multiple medical institutions over a 2-week capture period from 1 March 2022 until 14 March 2022 inclusive. Data collected included age, sex, injury, length of stay and dates of speciality input.

**Results:**

A total of 1,050 patients were included from 27 hospitals. The median age was 80 years, with 560 (53.3%) of all fractures being neck of femur fractures. Of the 1,050 patients, 870 (82.9%) were managed operatively. The median number of different speciality involvements was 3; 645 (61.4%) had an orthogeriatric (OG) review. In major trauma centres (MTC), 93.3% had OG input, compared with 66.3% in non-MTC. The speciality with the greatest input was Radiology, with Plastics having the lowest input.

**Conclusion:**

A standardised MDT approach is needed to optimise care and recovery in orthopaedic trauma patients. The difference in results regarding speciality involvement is substantial and needs to be addressed to minimise disparities in care received by this vulnerable cohort of patients.

## Introduction

Orthopaedic trauma accounts for approximately 63% of all orthopaedic referrals in the UK.^1^ The management of often complex injuries is a critical part of effective trauma care. The combination of an ageing population and increased demand on trauma services has led to new frameworks being implemented to achieve the best outcome for patients.^2^

A multidisciplinary team (MDT) approach to trauma has become popular due to improved outcomes involving specialists such as trauma surgeons, anaesthetists, radiologists, and orthogeriatric (OG) and emergency medicine doctors.^3^ An MDT is made up of clinicians and allied health professionals who work together to achieve shared care goals.^3^ This approach takes a holistic view of patient care, including fracture and injury management, rehabilitation and medical optimisation to help reduce length of stay. Coordinating safe, reliable healthcare requires effective teamwork to minimise mistakes.^4,5^ Failures in the MDT process can potentially lead to delays in treatment and increased length of stay, with poor communication and ineffective teamworking shown to be independent causes of preventable patient harm.^5^

Fragility fractures are a major public health problem associated with significant mortality and complications.^6^ Multiple studies have demonstrated the importance of early intervention in patients with fragility fractures, as it reduces complications and mortality.^7^ Previous studies have reported mortality rates of up to 10% during acute admissions of patients with hip fractures.^8,9^ Moja *et al* conducted a meta-analysis of 190,000 patients across 35 studies, finding that intervention before 48 hours significantly reduced mortality.^10^ A recent systematic review of 37 studies found that good OG care reduced length of stay, delirium and cost.^11^

An MDT approach has shown significant benefit for patients with fragility fractures, with management of patients presenting with neck of femur (NOF) fractures a common example.^12^ In this case, emergency doctors are responsible for the patient’s initial management, including fluid resuscitation, investigations, pain control and emergency medical care. Radiologists help with interpreting equivocal imaging, and advising regarding further investigations as necessary. Orthopaedic surgeons assess the patient and their injury and formulate an appropriate management plan. Anaesthetists ensure patient optimisation and safety throughout any surgical intervention. Following surgery, the geriatricians and/or Intensive Care physicians provide input into the management and care of the patient until discharge. The British Orthopaedic Association Standards of Trauma (BOAST) guidelines have helped standardise the MDT management of frail orthopaedic patients. However, unless trusts adhere to them, there will be a shortfall in the standard of care.^8^

The objective of this research was to assess to what extent the BOAST guidelines for the management of frail Orthopaedic patients are being met. The project aims to evaluate different speciality inputs for current trauma inpatients and identify whether there is a need for a more cohesive multidisciplinary engagement to support the care of the patient and staff.

## Methods

**Figure 1:**
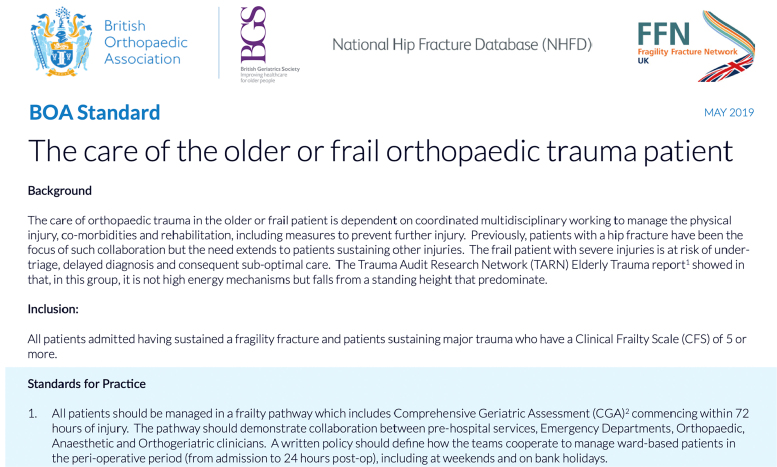
BOA standard for care of the older or frail orthopaedic patient.^12^ BOA = British Orthopaedic Association

This study was conducted against the British Orthopaedic Association standard ‘The care of the older or frail orthopaedic trauma patient (Figure 1).^13^ The Health Research Authority Decision tool was used to assess the need for research and Ethics Committee approval.^14^ Collaborators were recruited from multiple institutions in the UK, with registration of their involvement and ethical approval obtained at each local hospital as necessary.

A retrospective analysis was performed of all Trauma and Orthopaedic patients in the included institutions over a 2-week capture period from 1 March 2022 until 14 March 2022 inclusive. All inpatients during this 2-week period had their length of stay recorded, even if their date of admission was before the period or their date of discharge was after the period. Each subspeciality involved in the patients' care was recorded.

The inclusion criteria for this study were all adult orthopaedic trauma inpatients on 1 March, including polytrauma patients and those managed nonoperatively. All elective admissions were excluded. Fragility fractures were defined broadly as proximal and distal femur, radius/ulna, spine and humerus in patients over 65 years of age. Patients were further stratified during analysis as whether they were from major trauma centres (MTC) or not.

Data collected included age, sex, fracture location, type of speciality input and corresponding dates, surgical input and length of stay. Medical and surgical input were categorised by speciality, for example, anaesthetics.

### Analysis

Data were inputted and subsequently analysed using RPubs, version 3.6.2.^15^ RPubs is a publishing platform by RStudio – a programming and statistical computing integrated development environment.^16^

Data were summarised as descriptive statistics, including median, interquartile range (IQR) and percentages.

## Results

Data were collected from a total of 97 contributors across 27 hospital sites, 4 of which were MTCs. A total of 1,050 patients were included across the capture period. The primary orthopaedic injury was categorised based on the injury site or type; for example, soft tissue injuries accounted for 9.9% (105) overall. The median patient age was 80 years, with 663 (63.1%) female and 387 (36.9%) male patients. Baseline demographics including age, sex and type of injury are included in Table 1.

**Table 1:** Demographics of patients included divided between hip fracture groups and non hip fracture group

Characteristic	Overall, *N*=1,050	Hip fracture, *N*=560	Nonhipfracture,*N*=490
Sex			
Female, *n* (%)	663 (63)	381 (68)	282 (58)
Male, *n* (%)	387 (37)	179 (32)	208 (42)
Age, years (IQR) [range]	80 (67, 87) [18, 102]	83 (76, 88) [19, 102]	73 (54, 83) [18, 101]

IQR = interquartile range

Overall, the median number of specialities providing input was three.

**Figure 2:**
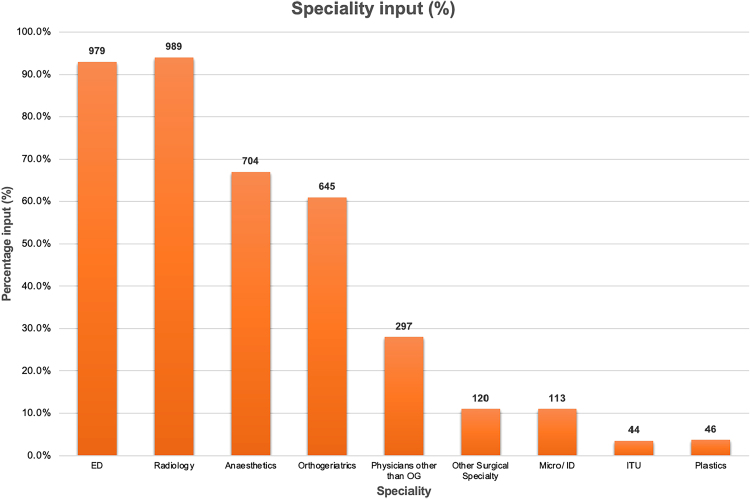
Graph demonstrating frequency of recorded speciality input (%)

Figure 2 outlines the contribution of each speciality to patient management. Radiology and Emergency Department accounted for the largest contributions, 994 (94%) and 983 (93%), respectively. The total number of probable fragility fractures was 437 (41.6%), of which 69.1% had documented OG input. Stratifying according to institution, 42/45 (93.3%) MTC patients had OG input. In non-MTC hospitals, 260/392 (66.3%) patients had OG input.

Table 2 demonstrates the total number of fractures and location. The table has been divided into hip fracture and nonhip fracture, with nonhip fracture representing 497 (47%) of the total patients included. There were 560 NOF fractures, accounting for 53% of all fractures recorded; 877 (83%) of all patients were managed operatively. The IQR of age for hip fracture patients was 76–88, whereas for nonhip fracture patients it was 54–83 years old. Table 3 presents the speciality input grouped by nonhip fracture and hip fracture.

**Table 2:** Fracture diagnosis and frequency

Fracture location	Overall, *N*=1,050	Hip fracture, *N*=560	Nonhip fracture, *N*=490
Acetabulum/pelvis	23 (2.2%)	0 (0%)	23 (4.7%)
Ankle	56 (5.3%)	0 (0%)	56 (11%)
Carpus/hand	5 (0.5%)	0 (0%)	5 (1.0%)
Clavicle	5 (0.5%)	0 (0%)	5 (1.0%)
Femur proximal	560 (53%)	560 (100%)	0 (0%)
Femur shaft or distal	56 (5.3%)	0 (0%)	56 (11%)
Humerus	31 (2.9%)	0 (0%)	31 (6.2%)
Other/polytrauma	78 (7.4%)	0 (0%)	78 (16%)
Other/soft tissue	105 (9.9%)	0 (0%)	105 (21%)
Patella	12 (1.1%)	0 (0%)	12 (2.4%)
Radius/ulna	9 (0.9%)	0 (0%)	9 (1.8%)
Scapula	1 (<0.1%)	0 (0%)	1 (0.2%)
Spine	42 (4.0%)	0 (0%)	42 (8.5%)
Talus or foot	9 (0.9%)	0 (0%)	9 (1.8%)
Tibia	58 (5.5%)	0 (0%)	58 (12%)

**Table 3:** Details of patient admissions and breakdown of speciality involvement in their care

Characteristic	Overall, *N*=1,050	Hip fracture, *N*=560	Nonhip fracture, N = 490
Length of stay, days (IQR) [range]	14 (9, 23) [0, 250]	15 (10, 24) [0, 250]	13 (7, 22) [0, 250]
Treated operatively	870 (83%)	547 (98%)	323 (66%)
Number of operations (IQR) [range]	1.0 (1.0, 1.0) [1.0, 9.0]	1.0 (1.0, 1.0) [1.0, 4.0]	1.0 (1.0, 2.0) [1.0, 9.0]
Number of specialties	4.00 (3.00, 5.00) [0.00, 10.00]	4.00 (4.00, 5.00) [1.00, 10.00]	3.00 (3.00, 4.00) [0.00, 10.00]
ED	979 (93%)	540 (96%)	439 (90%)
Radiology	989 (94%)	516 (92%)	473 (97%)
Anaesthetics	704 (67%)	437 (78%)	267 (54%)
OGs	645 (61%)	498 (89%)	147 (30%)
Physicians other than OG	297 (28%)	170 (30%)	127 (26%)
Other surgical specialty	120 (11%)	33 (5.9%)	87 (18%)
Micro/ID	113 (11%)	45 (8.0%)	75 (14%)
ITU	44 (3.5%)	8 (1.4%)	36 (7.2%)
Plastics	46 (3.7%)	1 (0.2%)	45 (9.1%)

ED = emergency department; Micro/ID = microsurgical/infectious diseases; IQR = interquartile range; ITU = intensive therapy unit; OG = orthogeriatric

**Figure 3:**
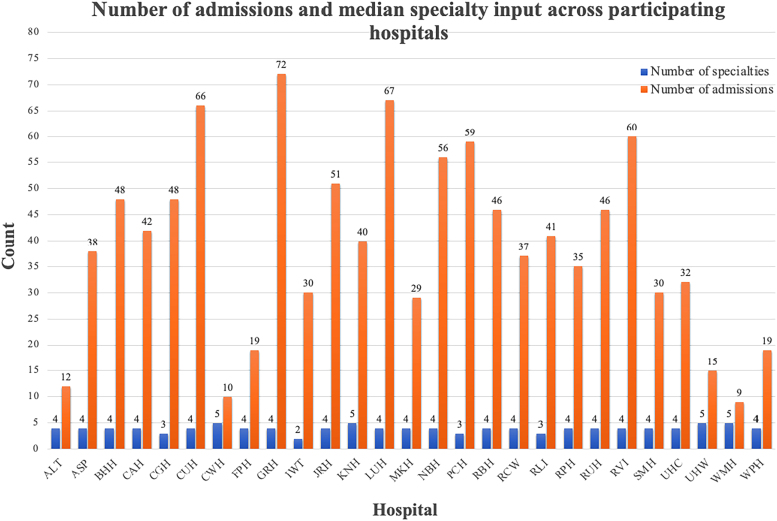
Graph showing number of admissions and speciality input by hospital

Figure 3 shows the number of admissions per site versus the number of different speciality inputs. Some hospitals had significantly more admissions, with the highest recorded being 72 admissions, and the lowest being 9 admissions. The highest number of different speciality involvement was five, with the lowest being two.

## Discussion

Orthopaedic trauma encompasses a broad range of soft tissue and bony injuries, often requiring input from multiple specialities to minimise morbidity. Hip fractures in particular are associated with significant morbidity and loss of independence.^17^ MDT collaboration can speed up preoperative assessment and subsequent management.^18^ A study conducted in 2019 looked at ways to minimise the length of stay by using Enhanced Recovery After Surgery, using a MDT approach to reduce postoperative complications. The study reported faster recovery, increased patient satisfaction and reduced length of stay.^19^

This study has highlighted that, despite published guidelines being in place, not all hospitals are adhering to them. For example, only 69.1% of patients with fragility fractures received OG input, when the guidelines state that ‘All patients should be managed in a frailty pathway which includes Comprehensive Geriatric Assessment (CGA) commencing within 72 hours of injury. The pathway should demonstrate collaboration between pre-hospital services, Emergency departments, Orthopaedics, Anaesthetics and Orthogeriatric clinicians’.^2,13^ The variation in clinical practice indicates further monitoring is required to improve outcomes and standards of care.

In the UK, there are an estimated 300,000 fragility fractures per year, with this figure increasing year on year.^20,21^ Ageing populations with multiple comorbidities make managing frail orthopaedic inpatients increasingly more complex, placing a significant burden on healthcare resources.^22^ NOF patients are particularly vulnerable, with a mortality of 12.6% at 30 days.^23^ Non-NOF patients had reduced speciality input on average compared with NOF patients in this study. The primary reason for this was that fewer non-NOF patients were managed operatively, therefore requiring less anaesthetic input.

Equitable distribution of healthcare resources can place a significant challenge for the NHS. Tariff payments are when trusts receive additional payments for hitting certain criteria such as surgical fixation within 36 hours.^24^ These are currently limited to only a few orthopaedic operations such as NOFs. However, the question remains: would expanding tariff payments lead to more timely MDT care? This may lead to care being financially driven, and individuals outside the tariff criteria receiving poorer care.

Annually, hip fractures account for 1.5 million inpatient bed days, with this figure expected to double by 2050.^25,26^ Effective MDT collaboration across specialities is crucial to ensure optimal patient care. A study conducted by Neuberger *et al* assessed the impact of increased OG input in management of hip fractures across 150 hospitals between 2010 and 2014. The study found that there was a 3.4% reduction in mortality at 30 days, which was attributed to increased MDT input.^27^

An interdisciplinary healthcare approach to the frail orthopaedic patient can help to optimise care and minimise mortality. Standardised care will reduce demand on ward doctors and staff, which can then ultimately aid guidelines being adhered to. The previous Getting It Right First Time initiative published in 2020 outlined the recommendations made after various reviews of orthopaedic elective practice.^28^

One area of improvement was length of stay for total hip replacements and total knee replacements. It was concluded that there was a reduction in bed resources by 20% between 2013/2014 and 2018/2019, which was attributed to factors such as increased training and guidance for physiotherapists to meet standards of care for recovery. Improved communication between hospitals in a trust ensured that equipment for low-volume, complex procedures was available more than 90% of the time. The improved teamwork reduces time to surgery, and also ensures that surgeons are not de-skilling in certain complex operations.

Regular MDT reviews will provide open discussion and allow staff to identify system gaps which may result in patient harm.^29^ Workplace challenges such as staffing issues, increased workload and dysfunctional teams can make effective MDT difficult.^30^ It is essential that there is regular review of practice and adherence to published guidelines to ensure good communication to enhance patient care.

Several limitations existed in this study. First, due to this study being a multicentre collaborative data collection, there was some discrepancy in the way data were recorded. This resulted in variation in perception of speciality input between hospital sites. The study period facilitated only a snapshot of speciality input, which may not necessarily be representative of overall input annually. Further staggered snapshot studies over longer periods of time should be considered in the future to be able to provide a more accurate representation.

Clinical frailty scales (CFS) could not be included in this study.^31^ For CFS to be calculated, the individuals recording data would need to have direct involvement with patient care or have the score documented in notes, which was not feasible in the majority of cases. CFS is also subjective and does not account for those with learning disabilities or dementia.^32^

## Conclusion

To optimise care and minimise morbidity, there is a need for standardised care in management of the frail orthopaedic patient. Patient-centred MDT care provides the best outcomes for the frail orthopaedic trauma patient, and further reviews into best practice should be conducted to ensure guidelines are met.
